# The dysfunctional host response to influenza A H7N9: a potential treatment option?

**DOI:** 10.1186/cc13839

**Published:** 2014-04-22

**Authors:** Steven M Opal, David S Fedson

**Affiliations:** 1 Department of Medicine, Alpert Medical School of Brown University, Memorial Hospital of RI, 111 Brewster Street, Pawtucket, RI 02860, USA; 2 57 chemin du Lavoir, 01630 Sergy Haut, France

## Abstract

The newly emerging human pathogen influenza A H7N9 represents a potentially major threat to human health. The virus was first shown to be pathogenic in humans in 2013, and outbreaks continue to occur in China to the present time. The current incident mortality rate is disturbingly high despite the frequent use of antiviral therapy and intensive care management. If the virus gains the capacity for efficient person-to-person transmission, a global influenza pandemic could ensue with devastating consequences. In the absence of an effective vaccine, targeted regulation of the host immune response by immune modulators might be considered. Readily available, approved drugs with immune-modulating activities might prove to be a treatment option in combination with existing antiviral agents and supportive care.

## 

In this issue of *Critical Care* Wu and colleagues provide a tantalizing glimpse into the immune dysfunction and possible immunopathogenesis of infection due to the novel H7N9 avian strain of influenza A virus [1]. Human cases of H7N9 influenza have thus far occurred only in China and surrounding regions [2-4], but there is disturbing evidence that this virus could easily evolve and create a pandemic of immense proportions. The observed case fatality rate in H7N9 influenza is much higher (about 25%) than that seen in patients infected with pandemic H1N1 virus in 2009 [5,6]. The H7N9 virus is derived from an avian influenza A virus that has crossed species barriers to infect humans, primarily those who have had exposure to live poultry markets. The virus spreads by respiratory aerosols, and limited human-to-human transmission probably occurs, although there is no evidence of continued spread thus far [2,3]. The virus causes essentially asymptomatic infection in chickens, but when transmitted to humans it leads to a highly symptomatic and potentially lethal illness. Severe H7N9 illness affects predominantly older patients (mean age ≥60 years), unlike pandemic H1N1 or avian influenza A H5H1 virus infections that affect younger individuals [1].

Wu and colleagues studied 27 H7N9 patients who were hospitalized at one center in Zhejiang Province in China and compared their immune responses with 30 healthy controls. All of the patients were severely ill (mean Acute Physiology and Chronic Health Evaluation II score 22) and all were treated with oseltamivir. Diffuse lymphopenia was seen in two-thirds of patients. More than 70% of patients developed acute respiratory distress syndrome and almost 50% acquired secondary bacterial pneumonia.

On the day of admission, the investigators performed multiplex cytokine analyses and flow cytometry to survey circulating peripheral blood mononuclear cells for activation or markers of T-cell anergy or exhaustion. Compared with healthy controls, cytokine analyses revealed elevated IL-6, IL-8 and IL-10 levels, but no increase in tumor necrosis factor, gamma-interferon or other proinflammatory cytokines. Flow cytometry showed increases in CD38, a marker of T-cell activation, and T-cell immunoglobulin and mucin protein-3 (TIM3), a surface marker for T-cell anergy/exhaustion. Conversely, human leukocyte antigen DR expression and TIM3-expressing monocytes were significantly lower. TIM3 is a well-characterized indicator of T-cell exhaustion in the mice, but this may not be true in humans [7]. Regrettably, the investigators did not measure programmed cell death-1 on T cells, which is a reliable marker of lymphocyte exhaustion in both mice and humans [1]. They were also unable to perform serial determinations of inflammatory markers to document the course of immune dysfunction over time.

The clinical and immunologic findings in H7N9 influenza differ substantially from those seen in younger patients infected by the pandemic H1N1 influenza virus or the more pathogenic avian influenza A H5N1 viruses [1]. H5N1 patients often have significant elevations of tumor necrosis factor and gamma-interferon, neither of which was seen with H7N9 influenza. Instead, a mixed pattern of proinflammatory (IL-6 and IL-8) and anti-inflammatory (IL-10) cytokines was observed, along with simultaneous expressions of T-cell activation (CD38) and de-activation (TIM3). These differences might reflect the unique virulence properties of the H7N9 virus or differences in the host response of older H7N9 patients.

A major question is whether patients with severe H7N9 influenza could benefit from immune modulator therapy in addition to antiviral agents and supportive care [8,9]. Many fundamental questions need to be answered. It is unclear whether the immune profile described by Wu and colleagues is a manifestation of the host response to the virus or whether immune dysregulation is actually contributing to the pathogenesis of the disease. Could the immune response be specifically targeted to aid patient recovery? Optimal treatment may not simply be a question of giving proinflammatory or anti-inflammatory agents. It may depend upon patient age, the specific virus, the presence of co-morbid illness, the extent of disease and the timing of the intervention [10-12].

Several commonly prescribed therapeutic agents that have immunomodulatory effects have been shown to be effective in treating influenza in experimental settings and in observational studies of patients with pneumonia and influenza (and even one randomized controlled trial in sepsis; Table 1) [9,13]. Statins are the agents best studied, but several other drug classes might also have clinical utility [13,14]. Many of these drugs are produced as inexpensive generics and are widely distributed in low-income and middle-income countries [9,13].

**Table 1:** Low-cost, readily available drugs with immunomodulatory activities that could benefit patients with severe influenza

**Drug class**	**Proposed mechanism of protection for influenza**	**Level of evidence**
HMG CoA reductase inhibitors (statins); lipid-lowering agents with numerous anti-inflammatory and vasoprotective effects	Alter prenylation pattern of enzymes and membranes: vaso-protection; lower TLR signaling; reduce oxidative stress; upregulate eNOS; upregulate AMPK and PGC-1 alpha; reduce NADPH oxidase activity	Animal studies; numerous observational studies in pneumonia and one in human influenza; one randomized, controlled trial in human sepsis
Aspirin, a cyclooxygenase inhibitor, might potentiate statin effects	Unknown, may shunt oxygenated lipid precursors to lipooxygenase pathway; might amplify statin actions	Observational studies in patients with severe pneumonia
PPAR-gamma agonists (thiazolidinediones, glitazones); anti-diabetic treatments with effects on mitochondrial biogenesis	Agonist for PGC-1 alpha that promotes mitochondrial function and biogenesis, might limit apoptosis	Animal studies with influenza virus challenge models
PPAR-alpha agonists (fibrates); lipid-lowering agents with effects on mitochondrial biogenesis	Promotes mitochondrial function, biogenesis	Animal studies with influenza virus challenge models
Angiotension II inhibitors – ARB agents and ACE inhibitors	Blocks angiotensin II ligation to AT-1, limiting superoxide generation by NADPH oxidase	Human observational studies in pneumonia patients, no data on influenza patients thus far
AMPK agonists (metformin), anti-diabetic agents with immune-metabolic effects	AMPK reduces oxidant stress and induces anti-apoptotic signals	Preclinical studies, no data in influenza patients thus far
Resveratrol, a polyphenol nutritional supplement	Stimulates sirtuin activity, activates PPAR alpha and PGC-1 alpha, protects against oxidant stress	Improved survival in animal studies with influenza challenge models

ACE, angiotensin-converting enzyme; AMPK, adenosine monophosphate protein kinase; ARB, angiotensin receptor blocker; AT-1, angiotensin II type 1 receptor; eNOS, endogenous endothelial nitric oxide synthase; HMG CoA, 3-hydroxy-3-methylglutaryl coenzyme A; NADPH, nicotinamide adenine dinucleotide phosphate hydrogen; PGC-1 alpha, peroxisome proliferator activated receptor gamma coactivator-1 alpha; PPAR, peroxisome proliferator activated receptor; TLR, toll like receptor.

Randomized controlled trials will be needed to determine whether adjuvant therapies will benefit patients with severe influenza. These studies should be conducted during outbreaks of seasonal influenza. We concur with health officials in the USA that it is morally imperative to perform such investigations before and during pandemics in order to guide clinicians responsible for managing patients with severe influenza [15]. Undertaking such trials during pandemics will take courage and coordination, but failure to act in an organized fashion will condemn patients to haphazard and unregulated efforts by clinicians desperate to find something that might benefit their patients. In the 21st century it is unacceptable to remain uninformed and ill prepared in inter-pandemic periods when we know that it is simply a matter of time before the next influenza pandemic occurs. The study by Wu and colleagues should prompt an international effort to better characterize the host response in patients with severe influenza and to design clinical trials that will define successful strategies for treatment.

## Abbreviations

IL: interleukin; TIM3: T-cell immunoglobulin and mucin protein-3.

## Competing interests

SMO has received funds from Sciclone and BioAegis for providing advice on the design of sepsis clinical trials. SMO is a consultant for Arsanis and Amplimmune, and has received grants from Asahi-Kasei and Epigenetics Ltd. SMO is also on clinical trial data monitoring committees for Tetraphas and Achaogen. DSF declares that he has no competing interests.
